# Nutrient Intake Is Insufficient among Senegalese Urban School Children and Adolescents: Results from Two 24 h Recalls in State Primary Schools in Dakar

**DOI:** 10.3390/nu8100650

**Published:** 2016-10-20

**Authors:** Marion Fiorentino, Edwige Landais, Guillaume Bastard, Alicia Carriquiry, Frank T. Wieringa, Jacques Berger

**Affiliations:** 1 UMR 204 Nutripass IRD-UM-SupAgro, Institut de Recherche pour le Développement, 911 Avenue d’Agropolis, Montpellier 34394, France; edwige.landais@ird.fr (E.L.); franck.wieringa@ird.fr (F.T.W.); jacques.berger@ird.fr (J.B.); 2 GRET, S/C IRD Hann Maristes, BP 1386, Dakar 18524, Senegal; g.bastard@geres.eu; 3 Department of Statistics, Iowa State University, Ames, IA 50011, USA; alicia@iastate.edu

**Keywords:** 24 h recall, nutrient intake, school children, adolescent, Senegal

## Abstract

Due to rapid urbanization and high food prices and in the absence of nutrition programs, school children from urban areas in West Africa often have insufficient and inadequate diet leading to nutrient deficiencies that affect their health and schooling performance. Acute malnutrition and micronutrient deficiencies are prevalent in children from primary state schools of Dakar (Senegal). The objectives of the present study were to assess the overall diet of these children, to report insufficient/excessive energy and nutrient intakes and to investigate association between insufficient nutrient intake and micronutrient deficiencies. Children attending urban state primary schools in the Dakar area were selected through a two-stage random cluster sampling (30 schools × 20 children). Dietary intake data were obtained from two 24 h recalls and blood samples were collected from 545 children (aged 5–17 years, 45% < 10 years, 53% girls) and adjusted for intra-individual variability to estimate nutrient usual intakes. Energy intake was insufficient and unbalanced with insufficient contribution of protein and excessive contribution of fat to global energy intake in one third of the children. Proportions of children with insufficient intake were: 100% for calcium, 100% for folic acid, 79% for vitamin A, 69% for zinc, 53% for vitamin C and 46% for iron. Insufficient iron and protein intake were risk factors for iron deficiency (odds ratio, OR 1.5, 2.2). Insufficient zinc intake and energy intake from protein were risk factors for zinc deficiency (OR 1.8, 3.0, 1.7, 2.9). Insufficient iron and vitamin C intake, and insufficient energy intake from protein were risk factors for marginal vitamin A status (OR 1.8, 1.8, 3.3). To address nutritional deficiencies associated with a diet deficient in energy, protein and micronutrients, nutrition education or school feeding programs are needed in urban primary schools of Senegal.

## 1. Introduction

Malnutrition is estimated to be highly prevalent in African school-aged children, with 20% suffering from stunting, 30% suffer from thinness, 10% are overweight, 30% have anemia, 30% are iron-deficient, 30% are iodine-deficient, 30% have vitamin A deficiency, and 50% are zinc-deficient [1]. Due to growth, cognition and educational achievements, nutrient requirements of school-aged children (5–9 years) are high [1]. Because of late school enrollment often occurring in low and middle income countries (LMIC) primary school age also includes early adolescence, a period when skeletal and brain growth spurts and sexual maturation dramatically increase nutrient requirements [1]. Early marriage, still common in the developing world [2], makes adolescent girls particularly vulnerable to undernutrition in case of childbearing [3,4]. Thus, nutrient deficiencies may be especially harmful for cognitive and physical development during school age and early adolescence (10–14 years). Although physiological status and health conditions like menarche or parasitosis have an influence on nutritional status, nutritional status is mainly related to nutrient intake. Thus, dietary intake data are crucial to understanding the role of the diet in nutritional deficiencies, and eventually to design effective nutritional interventions.

In LMIC, diet of school-aged children and adolescents is usually poor in fruits, vegetables and animal products, leading to inadequate protein and micronutrient intake [5]. Moreover, urban new lifestyles increase sedentary activities and consumption of high fat and sugar snacks and beverages that are often energy dense but micronutrient poor [5]. Although living in urban areas was known to improve dietary diversity and therefore nutritional status [6], some evidence showed that in poor urban settings micronutrient deficiencies are still prevalent [7] and can coexist with increasing overweightness. Indeed, in African urban areas, school-aged children spend more time out-of-home away from their caretaker supervision and consume more street food compared to their younger counterparts [8]. Thus, changes in food patterns due to the transition from preschool to school age can affect nutrient intakes especially when no school feeding programs are available. Consequently, populations of school-aged children and adolescents living in African urban areas may be at risk for double burden of malnutrition associated with inadequate dietary intake.

Recent data on nutrient intake adequacy and its implication for nutrition in primary school children living in African urban settings is scarce. No data on dietary intake of school-aged children and adolescents from Senegal were available [5]. The authors conducted a cross-sectional study investigating anthropometry, micronutrient status and dietary intake among a representative sample of 600 children attending state primary schools in Dakar, Senegal. Results from anthropometric and biochemical data were published elsewhere [9]. Chronic malnutrition was low but acute malnutrition was prevalent, with less than 5% of the children being stunted and 18% of children having BMI(Body-Mass-Index)-for-age < −2 *z*-scores. Overweightness and obesity were not prevalent in that population. Micronutrient status was poor with 36% of children suffering from marginal vitamin A status, 26% being zinc-deficient and 39% being iron-deficient.

The objectives of the present article were: (i) to estimate nutrient intake (ii) to report prevalence of insufficient/excessive nutrient intake overall and by age and gender groups; and (iii) to evaluate inadequate nutrient intake as risk factors for micronutrient deficiencies measured on blood samples.

## 2. Participants and Methods

### 2.1. Study Design and Participants

The study was a representative cross-sectional survey targeting children registered in state primary schools in the area of Dakar. Here, 600 school children were randomly selected through a two-stage random cluster sampling of children attending state primary schools in the Dakar area (30 schools × 20 children). Details of study design and sampling are provided elsewhere [9]. Adolescents were defined as equal or above 10 years [10].

### 2.2. Data Collection

The study was conducted over 30 school-days in February–March 2010 by trained interviewers. Two quantitative 24 h recalls (24 h) were conducted, at school, with three school days between repeated recalls. Children were asked to provide details on all foods and beverages consumed during the previous day. Rice and cereal gruels were quantified by weighing salted replicas to the nearest gram, using an electronic scale Terraillon^®^. The other foods were quantified using pictures of different portion size [11], household measures, standard units or purchase price. During the afternoon following the 24 h, mothers or caretakers were asked at home about recipes of each home-cooked food or beverage mentioned by the child during the 24 h. The amount of each ingredient was estimated in grams, household measures or price. Similarly, recipes of foods (snacks and beverages) sold inside or outside schools were recorded by interviewing vendors. During the second 24 h, anthropometric measurements (height and weight), blood and urine samples were collected to measure vitamin A, zinc, iron and iodine status. Methods of blood samples analysis and assessment of micronutrient deficiencies as well as methods and results of socioeconomic survey are provided elsewhere [9].

### 2.3. Data Management and Analysis

Data entry, including quality checks and validation by double entry of questionnaires, was performed with EpiData entry version 3.1 (EpiData Association, Odense, Denmark). A food composition table compiled from different food composition tables [12,13,14,15] was specifically developed for the study. Data management was performed using EpiData Analysis version 2.2 (EpiData Association, Odense, Denmark). Energy and nutrient intakes from repeated 24 h were adjusted for intra individual variability to obtain estimated usual intakes, using PC-Side (PC Software for Intake Distribution Estimation, ISU, 1997, Chicago, IL, USA) [16]. Estimated energy requirement (EER) were calculated using Schofield equations taking into account height, weight, age, and Physical Activity Level (PAL) for active and low active children [17]. Cut-offs used to estimate insufficient and excessive intakes are summarized in the Supplementary Materials Table S1 [18,19,20]. Prevalence of insufficient and excessive nutrient intake was estimated using PC-Side. Prevalence of insufficient nutrient intake was secondly calculated based on nutrient intake not adjusted for day-to-day variability and presented in the Supplementary Materials Figure S1. Crude odds ratios (OR) and odds ratios adjusted for age group and gender were calculated to measure the association between insufficient nutrient intake and micronutrient deficiencies.

### 2.4. Ethics

The protocol was approved by the ethical committee of the National Health Research of Senegal. The school directors informed parents of the selected children on the purpose and proceedings of the study. Written informed consent was obtained from all parents at the beginning of the study. Severely anemic participants received iron supplementation as treatment.

## 3. Results

Dietary intake was obtained for 599 participants aged 5–17 years. Fifty-four children identified as misreporters were excluded, leading to a final sample of 545 children. Of the sample, 52.6% were girls and 45.1% were < 10 years, resulting in 116 boys < 10 years, 130 girls < 10 years, 142 adolescent boys and 157 adolescent girls.

Most of children had insufficient energy intake, 7% had insufficient protein intake and 88% had insufficient fiber intake. None of them had carbohydrate intake below recommendation. Mean contribution to energy from carbohydrates, proteins, lipids, poly-unsaturated fatty acids (PUFAs) and saturated fatty acids (SFAs) were within the acceptable macronutrient distribution ranges (AMDRs) but the contribution of proteins and carbohydrates to energy was insufficient in 31% and 5% of children, respectively. No children had excessive contribution of protein and carbohydrates to energy, but respectively 32%, 21% and 14% of children had excessive contribution of lipids, SFAs, and PUFAs (Table 1). Iron and vitamin C intakes were insufficient for half of the children (Table 2) and zinc and vitamin A intakes were insufficient for more than two-thirds. Calcium and folic acid intakes were insufficient in all children. No micronutrient was consumed above their respective Upper Limits.

**Figure 1:**
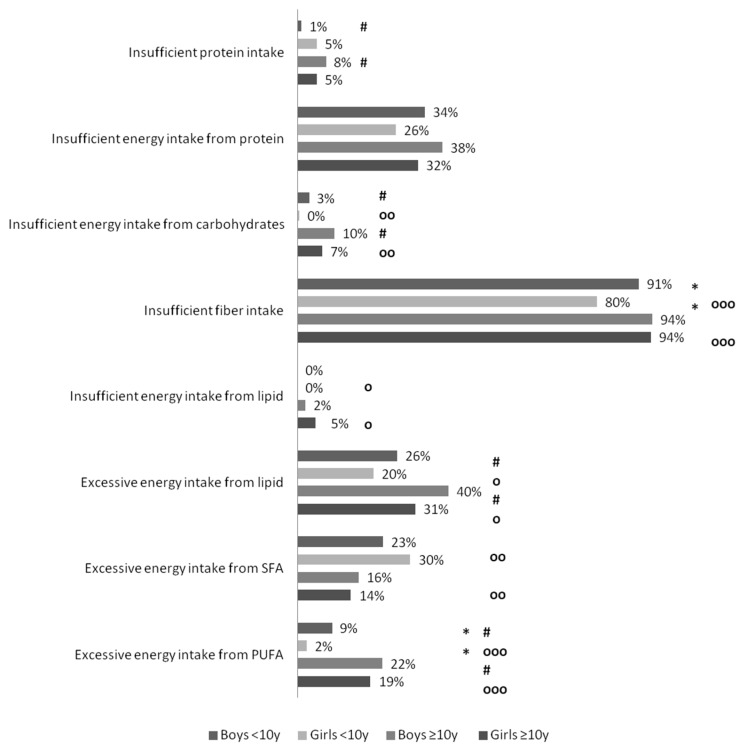
Prevalence of insufficient macronutrient intake according to age and gender group. %EI: Percent of energy intake; AMDR: Acceptable macronutrient distribution range; *p*-value for *χ*^2^-test between boys <10 years and girls <10 years; *: *p*-value < 0.05; *p*-value for *χ*^2^-test between boys <10 years and boys ≥10 years; #: *p*-value < 0.05; *p*-value for *χ*^2^-test between girls <10 years and girls ≥10 years: ooo: *p*-value < 0.001; oo: *p*-value < 0.01; o: *p*-value < 0.05.

**Figure 2:**
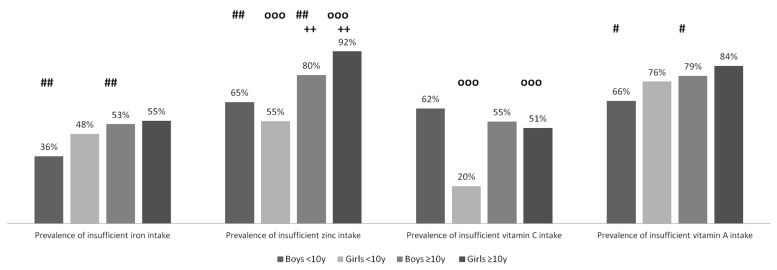
Prevalence of insufficient micronutrient intake according to age and gender group; *p*-value for *χ*^2^-test between boys ≥10 years and girls ≥10 years; ++: *p*-value < 0.01; *p*-value for *χ*^2^-test between boys <10 years and boys ≥10 years; ##: *p*-value < 0.01; # *p*-value < 0.05; *p*-value for *χ*^2^-test between girls <10 years and girls ≥10 years: ooo: *p*-value < 0.001.

Prevalence of inadequate nutrient intake and contribution of macronutrients to energy were different between age groups. Prevalence of insufficient contribution of carbohydrates to energy was higher in adolescents compared to children <10 years in both genders (Figure 1). Protein intake was lower in adolescents compared to children <10 years, among boys (1.17 ± 0.06 g/kg vs. 1.49 ± 0.08 g/kg, *p* < 0.001) and among girls (1.05 ± 0.05 g/kg vs. 1.41 ± 0.08 g/kg, *p* < 0.001). Insufficient protein intake was more prevalent among adolescent boys compared to boys <10 years (8% ± 5% vs. 1% ± 2%, *p* < 0.05). PUFA contribution to energy was higher in adolescents compared to children <10 years, in boys (13.3% vs. 12.3%, *p* < 0.001) and in girls (12.6% vs. 11.6%, *p* < 0.001), and so was prevalence of excessive contribution of lipid and PUFA to energy (Figure 1). Prevalence of excessive contribution of SFAs to energy was higher in girls <10 years compared to adolescent girls. Insufficient zinc intake was more prevalent in adolescents compared to children <10 years in both genders (Figure 2). Among girls, prevalence of insufficient vitamin C intake was higher in adolescents compared to girls <10 years (Figure 2). Among boys, prevalence of insufficient vitamin A intake was higher in adolescents compared to boys <10 years.

Differences in prevalence of inadequate intake according to gender were also observed. Intakes were higher in adolescent boys compared to adolescent girls in energy (1507 ± 45 kcal vs. 1399 ± 38 kcal, *p* < 0.001), in carbohydrates (193 ± 6 g vs. 183 ± 6 g, *p* < 0.05), in proteins (39 ± 2 g vs. 36 ± 1 g, 1.17 ± 0.06 g/kg vs. 1.05 ± 0.05 g/kg, *p* < 0.01 for both), in fibers (20 ± 1 g vs. 18 ± 1 g, *p* < 0.001), in lipids (58 ± 2 g vs. 53 ± 2 g, *p* < 0.001), in SFAs (14 ± 1 g vs. 13 ± 1 g, *p* < 0.05), and in PUFAs (23 ± 1 g vs. 20 ± 1 g, *p* < 0.001). Proportion of daily energy coming from PUFAs was higher in boys compared to girls, among children <10 years (12.3% ± 0.4% vs. 11.6% ± 0.3%, *p* < 0.001) and among adolescents (13.3% ± 0.4% vs. 12.6% ± 0.5%, *p* < 0.05). Excessive proportion of PUFAs in total energy was more prevalent in boys <10 years compared to girls <10 years (Figure 1). Mean vitamin C intake was lower in boys <10 years compared to girls <10 years (24 ± 2 mg vs. 34 ± 2 mg, *p* < 0.001). Mean folic acid and zinc intake were also lower in boys <10 years compared to girls <10 years (40 ± 2 µg vs. 47 ± 3 µg, *p* < 0.001; 4.6 ± 0.3 mg vs. 5.1 ± 0.3 mg, *p* < 0.05). Mean folic acid intake was higher in adolescent boys compared to adolescent girls (60 ± 3 µg vs. 51 ± 3 µg, *p* < 0.001). Prevalence of insufficient zinc intake was significantly higher in adolescent girls compared to adolescent boys (Figure 2).

The risk for iron deficiency was higher when intake of iron and protein were under estimated average requirement (EAR) and when intake were high in energy from lipids (Table 3). Zinc deficiency risk was higher when zinc intake was insufficient, when contribution of protein to energy intake was insufficient, when contribution of lipid and SFA was excessive and it was lower when fiber intake was below EAR. Risk for vitamin A marginal status was higher when iron intake, vitamin C intake and contribution of protein to energy intake was insufficient, and lower when energy intake from lipid was high.

## 4. Discussion

### 4.1. Energy, Macronutrients and Fiber Intake

Energy intake was insufficient in most children and the distribution of macronutrients was unbalanced with excessive lipid intake, especially from SFA, and insufficient protein intake for one third of the children. Few data on energy and macronutrient intake are available for African children but a study carried out in Cameroonian urban adolescents also indicated unbalanced diets with excessive carbohydrates and insufficient protein contributions to energy [21]. In the current study, lipid and SFA excessively contributed to energy intake and it contrasts with a study in Ghanaian rural school children where energy intake from carbohydrates and lipids were respectively higher and lower compared to the present study [22].

Most children had fiber intake below the adequate intake (AI). Even if fibers are not essential nutrients of which insufficient intakes lead to biochemical or clinical deficiency symptoms AI has been established to define fiber intake providing the greatest protection against coronary heart disease [23]. Moreover, dietary fiber is considered as protective against inflammatory bowel disease and colorectal cancer [24,25], of which excessive fat consumption, especially SFA, is suspected to be a risk factor [26].

Insufficient protein intake and contribution of protein to energy intake were respectively risk factors for iron and zinc deficiency. This is not surprising, as food rich in zinc and iron are usually also rich in protein (meat, shellfish, legumes), thus diets lacking of these products may lead to concomitant protein, iron and zinc poor status [27]. In the present study, excessive energy coming from lipids and from SFA were also risk factors for zinc deficiency, which is notable as some research suggests that the type of dietary fat influences the effects of zinc deficiency on fatty acid status and especially lipid concentrations in the liver [28]. We also observed that excessive lipid and PUFA intake was positively associated with iron deficiency. This could compensate the PUFA status decline induced by iron deficiency which is suggested in other research [29]. Indeed, Smuts et al assumed a possible disruption of PUFA metabolism in iron-deficient children when they observed higher SFA status and lower PUFA status among iron-deficient school children compared to children with normal iron status. Not surprisingly, in our study insufficient fiber intake was a protective factor for zinc deficiency, as dietary fiber is known to impair zinc absorption [23].

Even if energy intake increased with age, insufficient fiber, protein and micronutrient intake and inadequate contribution of macronutrients to total energy intake were higher among adolescents compared to children <10 years, which suggests poorer nutrition in adolescents, where mean height for age was lower in the present study [9]. A research conducted in Cameroonian urban adolescents also reported a high prevalence of insufficient contribution of proteins to energy intake [21]. These findings suggest that in African urban areas, the increase of macronutrient intake with age does not compensate drastically increased requirements at adolescence. Adolescent girls had the lowest ratio of energy intake by estimated energy requirement. Macronutrient intake and nutritional status of adolescent girls is particularly worrisome, as thinness of adolescent mothers is associated with low birth weight of their babies [1].

### 4.2. Iron Intake

In the present study, iron intake was poor, similar to what was reported in Ghanaian [22] and Kenyan school children [30]. Because of high consumption of tea and low meat intake among African urban children [31] the absorption of iron in the present population is probably low [32] and therefore prevalence of insufficient iron intake based on EARs may be underestimated. Although peers reported that iron intake does not predict well serum ferritin concentrations [33], in our study, insufficient iron intake was a risk for iron deficiency. Consequently, iron deficiency which affects 39% of school children of Dakar is likely to result from insufficient iron intake. By causing anemia and reducing physical and cognitive capacities, iron deficiency may impair school attendance and performance in the studied population [1]. Iron requirements are very high in adolescents especially in girls where menarche usually follows the growth spurt [32].

Insufficient iron intake was a risk factor for marginal vitamin A status. Some research suggested that iron deficiency may induce vitamin A deficiency by inhibiting mobilization of vitamin A store in rats [34] but the impact of iron deficiency on vitamin A metabolism remains unclear.

### 4.3. Zinc Intake

Insufficient zinc intake was highly prevalent in children, as reported in other studies in school children and adolescents from urban Cameroon [21], rural Ghana [22,35] and Kenya [22]. As reported in the literature [36], in the present study, insufficient zinc intake was a good predictor of serum zinc concentrations and therefore a good predictor of risk of zinc deficiency. Poor zinc intake and zinc deficiency may disturb growth, immune system and appetite and increase morbidity from diarrhea and acute respiratory infections [32]. Despite higher zinc intake, insufficient zinc intake was more prevalent in the adolescent groups compared to children <10 years, probably due to much higher requirements associated with growth spurt [32], where zinc has a central role [32].

### 4.4. Calcium Intake

In the present study, mean dietary calcium was below recommendations [23,37,38] and all children had insufficient calcium intake. Very high proportions of insufficient calcium intake were also reported in Cameroonian and Moroccan urban adolescents [21,39], and in school children in Ghana [22]. Dietary calcium has been shown to be correlated to serum calcium levels in African school children and adolescents [40] and low dietary calcium is associated with hypocalcaemia [41] and decreased bone mineral density [40]. So it is assumed that school children of Dakar are at high risk for calcium deficiency. Although rickets mostly affect infants, it can also occur during late childhood and adolescence if diet is poor in calcium or in vitamin D and/or high in phytates [42]. Therefore, although cut-offs used in the present study may be considered as too high, in other research on osteomalacia, mean levels of calcium intake in school children and adolescents suffering from rickets ranged from 150 to 300 mg, which is similar to the present study [42,43,44]. Hence the population in the present study is at high risk for calcium deficiency, and therefore for rickets and osteomalacia [45]. The insufficient intake of calcium in the present study may be surprising, as consumption of dairy products is culturally part of the Senegalese diet [46]. However their high price in urban areas reduces their accessibility.

### 4.5. Vitamin C Intake

Half of children had insufficient vitamin C intake. Elevated proportion of poor vitamin C intake was also observed in Kenyan and Ghanaian school children [22,30] as well as among Cameroonian and Nigerian adolescents [21,47]. As vitamin C intake was shown to be a good predictor of vitamin C serum values [36,48], school children from Dakar are likely to be at risk for vitamin C deficiency. A diet low in fruits rich in both vitamin A and vitamin C could explain why insufficient vitamin C intake was associated with poor vitamin A status in the present study [49]. Vitamin C deficiency results in skeletal pain, increased susceptibility to infections, impaired wound healing at a moderate stage, and scurvy at a severe stage [32]. Vitamin C has antioxidant properties [50,51,52] and is known to improve immune response and to reduce the incidence of infectious diseases in children from developing countries [53]. It also stimulates iron absorption and metabolism, especially for non-heme iron [54,55]. High cost and instability during storage are the major obstacles to the use of vitamin C in nutritional intervention programs [56,57] but distributing fruits through school feeding programs in African urban areas may improve vitamin C, iron, and vitamin A status, especially in vegetables based diets [22].

### 4.6. Folic Acid Intake

In the present study, all children had folic acid insufficient intake and mean folic acid intake was lower than what was reported in rural Kenyan and Ghanaian school children [22,30] although diets in these populations were already considered as folic-acid deficient. No recent data on folic acid status in Senegalese populations was available but a high risk of folic acid deficiency is assumed in this population as folic acid intake predicts well serum folic acid concentrations [36,58]. Insufficient folic acid intake and folic acid deficiency may result in leucopenia, intestinal malabsorption, blood coagulation impairment, increased sensibility to infections [32], and macrocytic anemia, which is the second most common type of nutritional anemia, after iron deficiency [59]. Among girls from the present sample, folic acid intake did not increase at adolescence, in contrary to most of the other micronutrients, and was lower than in boys. Similarly, insufficient folic acid intake was found in more than three-quarters of urban adolescents in studies in Cameroon and Morocco [21,39]. Thus, low folic acid intake in adolescent girls from African urban areas is worrisome, due to its serious adverse consequences during pregnancy on the development of the fetus [32].

### 4.7. Vitamin A Intake

Children from the present sample had poor vitamin A intake. Vitamin A intake is highly dependent on seasons [60]. In Senegal, mangos are only available from April to August, while the present study was conducted from February to March. A high proportion of children with insufficient vitamin A intake was also reported outside the mango season in a study conducted in Ghana [22]. Only 3% of children in the current study were vitamin A deficient, which is considered as mild public health issue and negligible compared to other urban school children from West Africa [7]. However, both prevalence of marginal vitamin A status [9] and insufficient vitamin A intake suggested a risk for vitamin A deficiency, which is involved in morbidity linked to diarrhea and measles, in growth retardation [32] and in large part of blindness or severe visual impairment [61]. No significant difference between boys and girls in the proportion of insufficient vitamin A intake was observed but boys were more affected by marginal vitamin A status than girls [9], which is consistent with previous research [62]. As growth rates under 10 years are higher in boys than in girls [32], the higher requirement of vitamin A in boys may not be adequately taken into account in EARs before adolescence. Risk for vitamin A poor status is probably a condition appearing at school age, as more than 90% of Senegalese children under 5 years receive yearly vitamin A supplementation [63]. Percentage energy intake (EI) coming from lipids above AMDR was a protective factor for marginal vitamin A status. Indeed, an increased intake of dietary fat is likely to improve the vitamin A absorption [32].

### 4.8. Dietary and Environment Determinants

African urban school children and adolescents are supposed to be more at risk of being overweight [1] and to consume more meat, vegetables, cereals, milk products than their rural counterparts [6]. However, in our study, children had insufficient micronutrient and energy intake, especially from protein, and acute malnutrition and micronutrient deficiencies were prevalent, while no overweightness nor low chronic malnutrition was observed. This suggests adequate care and diet in early childhood and degradation of diet and nutritional status when reaching school age [9].

Thus, nutrient intake data as well as biochemical and anthropometric data from this study indicated recent energy, protein and micronutrients deprivation in children and adolescents from state primary schools in Dakar, most likely due to deficient and inadequate diet, lacking of quantity and diversity with minimum consumptions of animal foods and fruits and vegetables. Consequently, fat and SFA excessively contributes to the total dietary intake, partly because of consumption of processed food and street food rich in low-quality lipids or sugar and poor in protein [5]. Indeed, it is known that urban lifestyle, added to the food price crisis and global crisis in the 2000’s, led to a reduction in the number of meals consumed at home in favor of street food in urban settings [8]. Research suggests that in low-income households, meat is the food category most restricted in the children’s diet [64]. Moreover, reduced supervision by mothers and caretakers at school age, and increased autonomy for feeding may also partly explain the degradation of dietary intake and nutritional status all along late childhood and adolescence. Indeed, without any public school feeding program in urban areas, children reaching the school age are mostly self-catered, which may cause poor food habits. As in many developing countries, school lunches in Senegal are limited to areas of high food insecurity, in rural areas, or where the World Food Program provides school meals.

### 4.9. Possible Interventions

Because of its implication in impaired physical and cognitive development during school age and adolescence [1], undernutrition associated with insufficient energy, protein and micronutrients intake in children from Senegalese urban schools should be addressed. School may be an efficient framework for nutritional and food interventions such as school feeding, nutrition education, or supplementation. A non-negligible part of the foods consumed by children and adolescents in urban settings is street food, therefore nutritional education programs should encourage the purchase of fruits instead of low-quality snacks [8]. Indeed, in other African settings, nutrition-based curriculum offered at school was shown to improve dietary behavior [65]. Accessible and cheap dark green leafy vegetables and legumes are sufficient to ensure adequate folic acid status in poor populations [66]. Although bioavailability of vitamin A is much higher in animal products, these are also less affordable so carotenoids are usually the main source of vitamin A for economically deprived populations [32]. Consequently, traditional Senegalese recipes containing unrefined palm oil and green leafy vegetables should be promoted among mothers in order to improve vitamin A and folic acid status. In other low-income settings, studies have shown the importance of education and nutrition knowledge in parents to improve the diet of children [67,68]. In African settings, school feeding programs are shown to improve intake of energy [69] and micronutrients [5,22], such as calcium, vitamin C and vitamin A through the distribution of fruits or dairy products. Beneficial effects of fortified food distribution through school feeding programs on micronutrient status were reported [70,71]. Calcium supplementation showed positive effects on calcium bone acquisition of children accustomed to low-calcium diets [72,73] and on long-term bone mass growth [74].

### 4.10. Strengths and Limitations of the Study

24 h recall can be as efficient as weighted records [75] and it is more accurate than a food frequency questionnaire in evaluating nutrient intake among school age children as reported in a review [76], especially when it is repeated at least once [77]. It is statistically more efficient to increase the number of individuals than the number of days of recall, but at least more than one day is needed to estimate the intra-individual variability [78]. It is admitted that children from 7 to 8 years are able to recall food consumed during the previous 24 h recall but that for children under 7 years, parental help is needed [79]. However, dietary recalls collected from mothers can be inaccurate as out-of-home food intake is often omitted from mother’s recall [80]. Plus, in the present study, it was not possible to interview children along with their mothers because of their out-door morning activities but they were asked to check the recall of their child when the interviewers came to the household to collect recipes. Among low-income households, fluctuations in food supplies especially low before paydays makes diets highly variable [64], which was taken into account in the present study conducted over 30 days. In Senegal, home-cooked meals are usually eaten in a common plate which complicates the estimation of individual’s consumption of staple food. The use of salted replicas to estimate rice portions in the present study was likely to be more accurate than the number of handfuls, which was reported as not satisfactory in Senegalese young children [81]. For other food, the use of handfuls, household measures and photographs may have lacked of accuracy [79]. However, authors assume that the purchase price was a good indicator for children and mothers with limited budgets, likely to remember well the prices of street food and ingredients. The food composition table was derived from five tables, in which composition of prepared meals or snacks in Senegal was not available, so we gathered quantitative recipes for all households and street vendors. Home-cooked meal composition was specific to each child for each day, and the composition of snacks sold in the school or in the surroundings was specific to each school. Therefore, one of the strengths of the study is that nutrient composition of food was not approximated and that inter-households, intra-areas and day-to-day variability were taken into account.

Despite its infrequent use, the EAR [82] is still considered by some peers as the most relevant dietary reference intake method for assessing risk of insufficient nutrient intake at population level, which takes into account the variability for both the requirement and the usual intake [18]. This method limits over- and underestimation of the prevalence [83] as it is observed in the present study and in other research [82]. However, the EAR cut-point may not be adapted to energy intake and to iron intake, due to the high correlation between intakes and energy requirements, and to the high skewness of iron requirement distribution in menstruating women [84]. However, only 38 girls in the present sample had already reached menarche, representing 7% of the whole sample. Overall, the main recommendations for using the EAR cut-point method to evaluate adequacy of nutrient intake in a group was followed: minimum 100 individuals, adjusting for day-to-day variability and excluding under-reporters [85]. One may object that EAR, AI and UL (Upper Limit) used in the current study were based on the diet of healthy individuals from North America, which may not be adapted to African populations. However, the poor micronutrient status indicated by the biochemical data supports high prevalence of insufficient nutrient intake found in the present paper.

## 5. Conclusions

Diets were energy-deficient and unbalanced between poor protein and fiber and excessive fat and SFA. For all micronutrients, at least half of the children had insufficient intake, suggesting a diet poor in dairy products, meat, fruit and vegetables, with a special concern for zinc, vitamin A, folic acid and calcium. Nutrient intake inadequacy increases with age. These findings are in accordance with anthropometric and biochemical data indicating high rates of thinness and micronutrient deficiencies. Similarly to what was observed in other African rural and urban populations of school-aged children and adolescents, nutrition of school-aged children and adolescents from primary state schools of Dakar is worrisome, because of high nutrient needs related to growth spurt, puberty, school achievement or early pregnancies in adolescent girls. Reduction of familial care, increase of street food consumption, and conjectural food prices crisis may explain a poorer diet at school age compared to infancy, which seems to be aggravated at adolescence. The findings of the present study highlight the need for nutritional interventions in Senegalese urban schools such as school feeding programs and nutrition education.

## Figures and Tables

**Table 1:** Mean energy and macronutrient daily intake (adjusted for within-person variability) and prevalence of insufficient and excessive macronutrient daily intake.

	Mean Intake/Proportion of Inadequate Intake	±
Energy (kcal)	1365	23
Children with energy intake below EER1 (%)	99%	1%
Children with energy intake below EER2 (%)	93%	2%
Protein (g)	36	1
Protein/body weight (g/kg)	1.25	0.04
Children with insufficient protein intake (%)	7%	2%
% Energy from protein	11%	0%
Children with insufficient energy intake from protein (%)	31%	4%
Children with excessive energy intake from protein (%)	0%	0%
Carbohydrates (g)	176	3
Children with insufficient fiber intake (%)	0%	1%
% Energy from carbohydrates	53%	0.4%
Children with insufficient energy intake from carbohydrates (%)	5%	1.8%
Children with excessive energy intake from carbohydrates% (%)	0%	0.3%
Dietary fiber (g)	19	1
Children with insufficient fiber intake (%)	88%	3%
Lipid (g)	52	1
% Energy from lipid	33%	0.3%
Children with insufficient energy intake from lipid (%)	1%	0.8%
Children with excessive energy intake from lipid (%)	32%	3.9%
SFA (g)	13	0
% Energy from SFA	8%	0%
Children with excessive energy from SFA (%)	21%	3%
MUFA (g)	15	0
% Energy from MUFA	9%	0.1%
PUFA (g)	19	0
% Energy from PUFA	12%	0.2%
Children with insufficient energy intake from PUFA (%)	0%	0.0%
Children with excessive energy intake from PUFA (%)	14%	2.9%

EER1: Estimated energy requirement for active children; EER2: Estimated energy requirement for low active children; SFA: Saturated fatty acids; MUFA: Mono-unsaturated fatty acids; PUFA: Poly-unsaturated fatty acids.

**Table 2:** Mean micronutrient daily intake (adjusted for within-person variability) and prevalence of insufficient and excessive micronutrient daily intake.

	Mean Intake/Proportion of Inadequate Intake	±
Iron (mg)	5.6	0.1
Children with insufficient iron intake (%)	46%	4%
Children with excessive iron intake (%)	0%	0%
Zinc (mg)	5.3	0.2
Children with insufficient zinc intake (%)	69%	4%
Children with excessive zinc intake (%)	1%	1%
Calcium (mg)	268	8
Children with insufficient calcium intake (%)	100%	0%
Children with excessive calcium intake (%)	0%	0%
Vitamin C (mg)	38	2
Children with insufficient vitamin C intake (%)	53%	4%
Children with excessive vitamin C intake (%)	0%	0%
Folic acid (µg)	50	2
Children with insufficient folic acid (%)	100%	0%
Children with excessive folic acid intake (%)	0%	0%
Vitamin A (µg)	309	17
Children with insufficient vitamin A intake (%)	79%	3%
Children with excessive vitamin A intake (%)	0%	0%

**Table 3:** Crude and age-gender adjusted odds ratios (OR) and confidence interval (CI) for insufficient nutrient intake associated with micronutrient deficiencies.

		Iron Deficiency		Marginal Vitamin A Status		Zinc Deficiency	
		OR	95% CI	OR	95% CI	OR	95% CI
Iron intake below EAR	Crude	1.32	0.93; 1.88	1.84	1.26; 2.68	1.05	0.74; 1.48
	Adjusted *	1.47	1.02; 2.13	1.80	1.22; 2.65	1.35	0.93; 1.96
Zinc intake below EAR	Crude	1.24	0.83; 1.84	1.53	1; 2.33	1.24	0.84; 1.82
	Adjusted *	1.46	0.95; 2.26	1.27	0.8; 2.02	1.76	1.13; 2.72
Vitamin C intake below EAR	Crude	0.74	0.52; 1.06	1.93	1.32; 2.83	1.24	0.87; 1.78
	Adjusted *	0.83	0.55; 1.24	1.80	1.21; 2.68	1.00	0.53; 1.87
Fiber intake below AI	Crude	0.68	0.39; 1.16	0.88	0.51; 1.5	0.09	0.05; 0.14
	Adjusted *	0.59	0.32; 1.11	1.13	0.64; 1.98	0.39	0.17; 0.88
Vitamin A intake below EAR	Crude	0.87	0.56; 1.35	1.73	1.07; 2.81	1.12	0.73; 1.72
	Adjusted *	0.72	0.43; 1.18	1.16	0.68; 1.96	1.20	0.73; 1.98
Protein per kg of body weight below EAR	Crude	2.20	1.17; 4.13	0.30	0.13; 0.67	0.36	0.12; 1.07
	Adjusted *	2.22	1.14; 4.33	0.44	0.19; 1.03	0.61	0.19; 2.02
% of energy coming from protein below AMDR	Crude	0.25	0.16; 0.39	4.58	3.03; 6.92	2.91	1.98; 4.29
	Adjusted *	0.34	0.22; 0.54	3.28	2.07; 5.19	3.00	1.91; 4.69
% of energy coming from carbohydrates above AMDR	Crude	0.17	0.08; 0.36	2.10	0.84; 5.3	4.26	2.47; 7.35
	Adjusted *	0.21	0.1; 0.45	1.87	0.69; 5.05	1.36	0.66; 2.81
% of energy coming from lipid above AMDR	Crude	2.04	1.41; 2.95	0.48	0.31; 0.75	1.93	1.33; 2.8
	Adjusted *	2.02	1.39; 2.93	0.47	0.29; 0.74	1.67	1.09; 2.56
% of energy coming from SFA above AMDR	Crude	1.44	0.91; 2.29	0.53	0.31; 0.92	3.31	2.04; 5.38
	Adjusted *	1.43	0.9; 2.28	0.57	0.32; 1	2.89	1.7; 4.91
% of energy coming from PUFA above AMDR	Crude	2.90	1.79; 4.68	0.37	0.19; 0.69	0.93	0.58; 1.49
	Adjusted *	3.02	1.85; 4.91	0.51	0.26; 1	0.99	0.59; 1.66

* Adjusted for age group and gender; AI: Adequate intake; AMDR: Acceptable macronutrient distribution ranges; EAR: Estimated average requirement; PUFA: Polyunsaturated fatty acid; SFA: Saturated fatty acid.

## Supplementary Materials

The following are available online at http://www.mdpi.com/2072-6643/8/10/650/s1, Figure S1: Unadjusted and adjusted prevalence of insufficient and excessive intake, Table S1: Recommendations of daily intake of macronutrients and micronutrients for populations of children 4–18 years.
