# Supplementary material for: Nutrient Intake Is Insufficient among Senegalese Urban School Children and Adolescents: Results from Two 24 h Recalls in State Primary Schools in Dakar
Source: Nutrients. 2016 Oct 20;8(10):650. doi: 10.3390/nu8100650 (PMC5084037; doi:10.3390/nu8100650)
Supplement: Supplementary file 1 [file nutrients-08-00650-s001.docx]

Supplementary Materials: Nutrient Intake Is Insufficient among Senegalese Urban School Children and Adolescents: Results from Two 24 h Recalls in State Primary Schools in Dakar

Marion Fiorentino, Edwige Landais, Guillaume Bastard, Alicia Carriquiry, Frank T. Wieringa and Jacques Berger

**Table S1.** Recommendations of daily intake of macronutrients and micronutrients for populations of children 4–18 years.

|  | **Vitamin A** | **Folic Acid** | **Vitamin C** | **Zinc** | **Iron** | **Calcium** | **Carbohydrate** | | **Protein** | | **Lipid** | **SFA** | **MUFA** | **PUFA** | **Fiber** |
| --- | --- | --- | --- | --- | --- | --- | --- | --- | --- | --- | --- | --- | --- | --- | --- |
| **Units** | **μg** | **μg** | **mg** | **mg** | **mg** | **mg** | **g** | **%EI** | **g/kg** | **%EI** | **%EI** | **%EI** | **%EI** | **%EI** | **g** |
| Insufficient intake | | | | | | | | | | | | | | | |
| Cut-off | EAR | EAR | EAR | EAR | EAR | AI | EAR | AMDR | EAR | AMDR | AMDR | - | - | - | AI |
| 4–8 years | 275 | 160 | 22 | 4 | 4.1 | 800 | 100 | 45 | 0.76 | 10 | 25 | - | - | 5 * | 25 |
| 9–13 years boys | 445 | 250 | 39 | 7 | 5.9 | 1300 | 100 |  | 0.76 |  |  |  |  |  | 31 |
| 9–13 years girls | 420 | 250 | 39 | 7 | 5.7 | 1300 | 100 |  | 0.76 |  |  |  |  |  | 26 |
| 14–18 years boys | 630 | 330 | 63 | 8.5 | 7.7 | 1300 | 100 |  | 0.73 |  |  |  |  |  | 38 |
| 14–18 years girls | 485 | 330 | 56 | 7.3 | 7.9 | 1300 | 100 |  | 0.71 |  |  |  |  |  | 26 |
| Excessive intake | | | | | | | | | | | | | | | |
| Cut-off | UL | UL | UL | UL | UL | UL | - | AMDR | - | AMDR | AMDR | - | - | - | - |
| 4–8 years | 900 | 400 | 650 | 12 | 40 | 2500 | - | 65 | - | 30 | 35 | 10 * | within limits of total fat * | 15 * | - |
| 9–13 years | 1700 | 600 | 1200 | 23 | 40 | 2500 |  |  |  |  |  |  |  |  |  |
| 14–18 years | 2800 | 800 | 1800 | 34 | 45 | 2500 |  |  |  |  |  |  |  |  |  |

These cut-offs are recommended in the essential guide to nutrient requirements of the Institute of Medicine (IOM) [18] except cut-offs for fatty acids intake * established by other peers [19]. SFA: Saturated fatty acid; MUFA: Monounsaturated fatty acid; PUFA: Polyunsaturated fatty acid; %EI: % of energy intake; EAR: Estimated average requirement; AI: Adequate Intake; AMDR: Acceptable macronutrient distribution ranges; UL: Upper limit; NB: In the present study, 167 (31%) participants were <9 years, 160 (29%) participants were boys aged from 9 to 13 years, 182 (33%) participants were girls aged from 9 to 13 years, 19 (3%) participants were boys ≥14 years, 17 (3%) participants were girls ≥14 years.


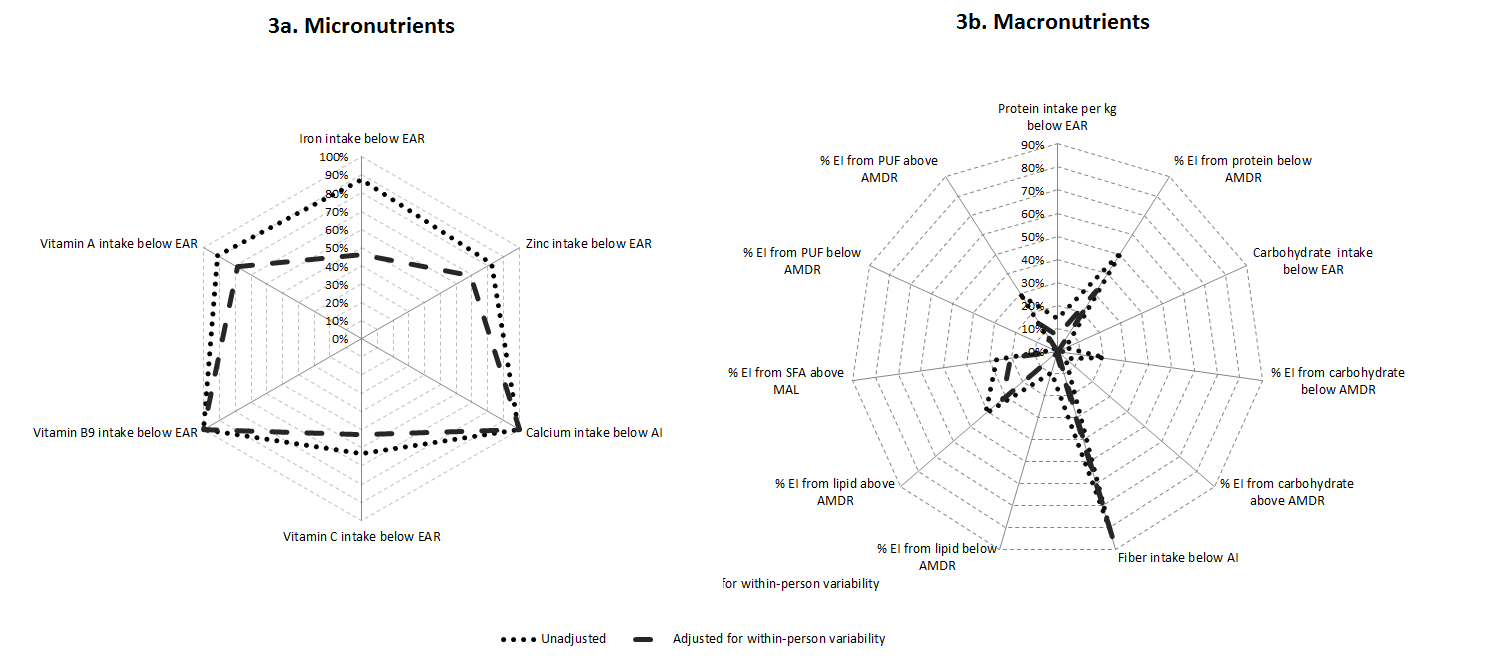


**Figure S1.** Unadjusted and adjusted prevalence of insufficient and excessive intake. %EI: % of energy intake; AI: Adequate intake; AMDR: Acceptable macronutrient distribution ranges; EAR: Estimated average requirement; PUFA: Polyunsaturated fatty acid; SFA: Saturated fatty acid; UL: Upper limit.
